# Comprehensive analysis of clinical prognosis and biological significance of CNIH4 in cervical cancer

**DOI:** 10.1002/cam4.6734

**Published:** 2023-12-12

**Authors:** Jiajia Wang, Shudan Wang, Junli Wang, Jingjing Huang, Haishan Lu, Bin Pan, Hanyi Pan, Yanlun Song, Qianqian Deng, Xiaojun Jin, Guiling Shi

**Affiliations:** ^1^ Department of Obstetrics and Gynecology The Affiliated Hospital of Youjiang Medical University for Nationalities Baise China; ^2^ Industrial College of Biomedicine and Health Industry Youjiang Medical University for Nationalities Baise China; ^3^ School of Medicine Ningbo University Ningbo China; ^4^ School of Medical Laboratory Youjiang Medical University for Nationalities Baise China; ^5^ Clinical Pathological Diagnosis & Research Centra The Affiliated Hospital of Youjiang Medical University for Nationalities Baise China; ^6^ Department of Laboratory Animal Center Youjiang Medical University for Nationalities Baise China

**Keywords:** cervical cancer, cornichon homolog 4, immune landscape, knockdown, predictive model

## Abstract

**Background:**

Cornichon homolog 4 (CNIH4) belongs to the CNIH family. It functions as an oncogene in many tumors. However, CNIH4's significance in the immune landscape and its predictive potential in cervical cancer (CESC) is unexplored.

**Methods:**

CNIH4 levels and its effect on the survival of patients with CESC were evaluated using data retrieved from The Cancer Genome Atlas (TCGA). The oncogenic effect of CNIH4 in CESC was determined using small interfering RNA‐mediated transfected cell lines and tumorigenesis experiments in animal models.

**Results:**

Higher expression of CNIH4 was found in advanced tumor and pathological stages, as well as lymph node metastasis. CNIH4 expression correlated positively with the infiltration of macrophages M2 and resting dendritic cells into the affected tissue. Additionally, functional enrichment of RNA‐sequencing of CNIH4‐knocked down CESC cell lines showed the association of CNIH4 to the PI3K‐Akt signaling pathway. Single‐sample gene set enrichment analysis highlighted several immune pathways that were elevated in the CESC samples with enhanced levels of CNIH4, including Type‐I and Type‐II IFN‐response pathways. The impact of CNIH4 on drug sensitivity was further assessed using the GDSC database. As CNIH4 is linked to the immune landscape in CESC, this study determined a four‐gene risk prediction signature utilizing CNIH4‐related immunomodulators. The risk score quantified from the prediction signature was an independent predictive indicator in CESC. Receiver operating characteristic curve analysis verified the good predictive ability of the four‐gene signature in TCGA‐CESC cohort. Thus, the CNIH4‐related model showed potential as an auxiliary TNM staging system tool.

**Conclusion:**

CNIH4 may be an effective predictive biomarker for patients with cervical cancer, thus providing new ideas and research directions for CESC.

## 1 INTRODUCTION

Cervical cancer (CESC) ranks fourth worldwide in terms of morbidity and mortality rates. It is a common cause of cancer deaths and pathogenesis globally, with 338,800 new deaths (7.7%) and 598,000 new cases (6.5%) in 2020 alone.^1^ Available treatments for CESC include surgery, radiotherapy, and chemotherapy. However, they exert significant adverse effects and show unsatisfactory treatment outcomes. The prognosis of these patients is very poor, with a 5‐year survival rate of a mere 17%.^2^ Disease occurrence and progression of CESC result from diverse genetic and environmental factors due to CESC's molecular heterogeneity. An in‐depth detection of the key molecules and the associated mechanisms is significant for improved comprehension of the mechanisms underlying CESC tumorigenesis.

Furthermore, survival prediction and treatment of patients with CESC mainly depend on the TNM staging system which is mainly based on the tumor size, lymph node invasion, and distant metastasis to predict the prognosis of these patients. The traditional TNM classification prediction system only considers tumor volumes and ignores the effect of intratumoral molecular heterogeneity. This, however, may lead to differing clinical results even among patients at the same tumor stage and those receiving similar treatment regimens. This situation highlights the inadequacy of the present staging system in accurately predicting prognosis and making clinical decisions. Identifying key molecules associated with prognosis and diagnosis through tumor cell genomic analysis can improve the currently used TNM staging system, facilitating better prediction of clinical outcomes, personalized treatment options, and the development of novel therapies for patients with CESC.

Cornichon homolog 4 (CNIH4) belongs to the CNIH family. It is located on the long arm of Chromosome 1, Region 4, Band 2, and sub‐band.^3^, ^4^ Multiple studies show that CNIH4 is expressed in many malignant tumors and participates in their development, invasion, and metastasis.^5^, ^6^, ^7^, ^8^ Therefore, CNIH4 may function as an oncogene. Mishra et al. reported that TMED9 protein inhibits the enhancing effect of TMED3‐WNT‐TCF on colon cancer cell metastasis by promoting the CNIH4/TGF‐α/GLI cascade.^9^ Wang et al. reported a link between the expression of CNIH4 and the survival rate of patients with liver cancer, suggesting that CNIH4 may have diverse roles in different malignancies.^10^ The prognostic value and pathophysiological role of CNIH4 in CESC, however, remain unclear.

This study aimed to assess the expression, prognostic significance, and molecular mechanisms underlying CNIH4 in CESC patients using bioinformatics. The obtained data revealed possible functions of CNIH4 in CESC progression. We generated CESC cell lines with small interfering RNA (siRNA)‐mediated knockdown of CNIH4. The oncogenic characteristics of CNIH4 were further investigated in nude mice with xenograft tumors. The findings could help deepen the understanding of potential molecular pathology mechanisms and establish a basis for developing treatment strategies for CESC.

## 2 MATERIALS AND METHODS

### 2.1 Data collection

RNA‐sequencing (Seq) data (FPKM format) along with the clinical data of patients with CESC were retrieved from The Cancer Genome Atlas (TCGA). RNA sequencing data included 306 cases of CESC and 5 of paracancerous tissues. Ethical approval and informed consent were waived off for this study as the information from TCGA is publicly accessible.

### 2.2 Expression of CNIH4 in CESC and its association with prognosis and clinicopathological characteristics

The “limma” package was employed to evaluate the gene expression profile of CNIH4 in tumor and paracancerous tissues from TCGA‐CESC cohort. Based on the median gene expression of CNIH4 in TCGA‐CESC, 306 individuals were partitioned into two groups: high and low. The differences in overall survival (OS) and progression‐free survival between groups were compared using the “survival,” “limma,” and “survminer” packages. The link between CNIH4 gene expression and clinicopathological characteristics of CESC was explored. To determine independent factors impacting OS in patients with CESC (whose data were retrieved from TCGA), univariable and multivariable Cox proportional hazards regression analyses were utilized. The expression characteristics of CNIH4 at the single‐cell level were explored using the data for CESC in TISCH database and visualized on an umap reduced dimensionality plot.

### 2.3 Cell culture and siRNA transfection

Human cervical cancer cells, SiHa and Me180, were procured from Baioujing Biotechnology Co., LTD and cultured in the DMEM and MCESCoys 5A containing 10% fetal bovine serum, respectively. siRNA constructs for CNIH4 were purchased from Guangzhou RiboBio and transfected using Lipofactamine 2000 (Thermo Fisher Scientific, Waltham, MA, USA). 5′‐GGTTCATCTTCCTTCTCAA′ (siCNIH4‐1), 5′‐GTTGCCACTTGGAATATAT‐3′ (siCNIH4‐2), and 5′‐ CCTGTTGCCACTTGGAATA‐3′ (siCNIH4‐3) were the three target sequences of siRNA for CNIH4.

### 2.4 Establishing a nude mouse model of cervical cancer

BALB/c‐nude female mice were purchased from Beijing Vital River Laboratory Animal Technology Co., Ltd. After a week of acclimatization, the transfected SiHa and Me180 cells were digested and obtained as a 5 × 10^6^/mL single cell suspension and injected into the left armpit of BALB/c‐nude female mice with a 1 mL syringe following disinfection. After subcutaneous solid tumors were formed in the mice, the animals were sacrificed and the tumor tissues were excised and weighed.

### 2.5 Hematoxylin and eosin staining

Hematoxylin and eosin (HE) staining to observe the histological changes of tumor tissues. After weighing the tumor‐bearing tissues of nude mice, the tumor tissues or tissues of patients with cervical cancer were placed in 10% neutral formaldehyde, fixed at 4°C for 48 h, dehydrated by gradient concentration of ethanol, transparent in xylene, embedded in paraffin wax, cut into sections with a thickness of 4 μm, stained with hematoxylin and eosin in the conventional way, dehydrated, transparent, sealed, and observed the tumor histological morphology under a light microscope.

### 2.6 Immunohistochemistry

The specimens were dewaxed, hydrated, and thermally repaired. The samples were incubated in 3% H_2_O_2_ for 10 min and goat serum at room temperature for 30 min. Primary antibody, CNIH4 (1:400, invitrogen), incubation was conducted overnight at 4°C, followed by secondary antibody incubation at room temperature for 30 min. Diaminobenzidine was used to develop color. Finally, the specimens were dehydrated, made transparent, and sealed.

### 2.7 Western blotting

Cells were lysed with RIPA lysis buffer. Proteins were subjected to SDS‐PAGE. Separated proteins were transferred onto PVDF membranes. The membranes were blocked with 5% skim milk powder for 2 h. Membranes were subjected to incubation with primary antibodies against Tubulin (1:20000, Proteintech), BAX (1:2000, Proteintech), BCL‐2 (1:2000, Proteintech), CNIH4 (1:500, invitrogen), p‐PI3K (1:1000, arigobio biolaboratories), PI3K (1:1000, Hangzhou Huaan Biotechnology Co., Ltd), p‐AKT (1:1000, Proteintech), and AKT (1:1000, Proteintech) overnight at 4°C followed by secondary antibodies at room temperature for 2 h. Bands were detected with an ECL chromogenic solution, and Tubulin was used as a control for quantification.

### 2.8 Cell proliferation assay

Cervical cancer cells were grown in 96‐well plates at a density of 5 × 10^3^ cells per well. At the indicated time points of 0, 1, 2, 3, and 4 days post‐transfection, 10 μL of CCK‐8 solution was gradually added to each well with a row gun against the wall for 2 h. The optical density at 450 nm was measured.

### 2.9 Transwell assay

First, Transwell chambers with 8 μm wells were incubated for 4 h with Matrigel. In the lower chamber of a 24‐well plate, 600 μL of culture medium containing 10% serum was added, and 200 μL of SiHa or Me180 cell suspension was inoculated in the upper chamber. After 24 h incubation, the cells were fixed, stained, and counted under an inverted microscope.

### 2.10 Wound‐healing assay

SiHa or Me180 cells were inoculated in 6‐well plates. When the density reached ≥90%, three scratches were made in the 6‐well plate with a sterile 200 μL gun along a sterile straightedge and incubated in a medium without serum. Cells were observed and photographed under an inverted microscope after 0 and 24 h of scratching.

### 2.11 Functional enrichment analysis

RNA was extracted from 100,000 knockdown or wild‐type SiHa cells. The SMART technology (Clontech) was used to construct RNA‐Seq libraries. RNA‐Seq was performed using the illumine Nova‐Seq 6000 System. The GENCODE database was used to annotate mRNAs. Differentially expressed genes (DEGs) between the CNIH4‐knockdown and wild‐type SiHa cell lines were screened using the absolute value of logFC >0.5 and *p*‐value < 0.05 cutoff in the “limma” package. DEGs were subsequently subjected to Gene Ontology (GO) and Kyoto Encyclopedia Genes and Genomes (KEGG) assessment using the “clusterProfiler” and “org.Hs.eg.db” packages. The variance in the enrichment scores of the 13 immune‐related pathways (retrieved from previous studies) between both expression groups was measured by single‐sample gene set enrichment analysis (ssGSEA) utilizing the “GSVA” and “GSEABase” packages. GSEA was utilized to identify upregulated pathways related to elevated CNIH4 levels.

### 2.12 Association between CNIH4 expression and intratumoral immune cell infiltration

The cell‐type identification by estimating relative subsets of RNA transcript (CIBERSORT) analysis is an algorithm that utilizes gene expression signatures to predict the relative proportions of 22 different immune cell types. The variance between the high‐ and low‐CHIH4 expression groups with tumor‐infiltrating immune cell (TIIC) proportion was estimated using CIBERSORT tools (https://cibersort.stanford.edu/runcibersort.php). Subsequently, results were screened using *p* < 0.05. Using the Pearson correlation under “corrplot” package, the link between the levels of CNIH4 and the proportion of TIICs was assessed. Furthermore, the association between CHIH4 and the landscape of CESC gene mutations was explored using the CAMOIP online website (https://www.camoip.net) which provides a tool for analyzing the mutation data of TCGA. The differences between groups for the top 20 gene mutations from TCGA‐CESC were compared.

### 2.13 Prediction of drug sensitivity

The predictive value of CNIH4 gene expression for chemotherapy and targeted drug response was assessed using the “prrophetic” and “ggplot2” packages and the Genomics of Drug Sensitivity in Cancer database (https://www.cancerrxgene.org/). The half‐maximal inhibitory concentration (IC50) values represent the chemosensitivity between the two expression groups of TCGA‐CESC. A lower IC50 value indicates better drug therapy sensitivity.

### 2.14 Construction of the predictive model based on CNIH4‐related immunomodulators

CNIH4‐related immunomodulators (immunostimulators and immunoinhibitors) in CESC from the TISIDB website were used as the signature to build the CNIH4‐related prediction model for predicting OS. After obtaining the immunomodulators significantly associated with CNIH4, the variables related to the OS of patients with CESC were selected by univariate Cox analysis. Prognostic variables identified by univariate Cox analysis were subjected to further evaluation through a multivariate Cox analysis. A stepwise forward variable screening method was employed, and the “stepAIC” algorithm in the “MASS” package was employed to establish the predictive model.

The risk scores of each individual in the TCGA‐CESC cohort were quantified following the establishment of the model from the equation:

$$\begin{array}{c} \text{Risk score}=\text{gene}\;1\;\text{expression}\times \text{gene}\;1\;\text{coefficient}\\ +\text{gene}\;2\;\text{expression}\times \text{gene}\;2\;\text{coefficient}\\ +\ldots +\text{gene}\;n\;\text{expression}\times \text{gene}\;n\;\text{coefficient}. \end{array}$$

The optimal cutoff value of the risk score of the TCGA‐CESC cohort was obtained by utilizing the X‐Tile software (version 3.6.1). According to the cutoff value, the individuals in the TCGA‐CESC cohort were partitioned into high‐ and low‐risk groups (High‐risk group: 88 cases; low‐risk group: 216 cases).

### 2.15 Evaluating the model's predictive effect

Survival differences between both risk groups were evaluated utilizing Kaplan–Meier curves and log‐rank tests. To check the predictive ability of the models for 1‐, 3‐, and 5‐year OS and compare it with the TNM model (including staging and grading), the area under the curve (AUC) of the receiver operating characteristic (ROC) curve of the individual was measured using the “timeROC” package. To determine whether the CNIH4‐related model had better predictive accuracy for long‐term survival prediction, ROC curve analysis was employed.

### 2.16 Statistical analysis

The R language (4.2.1) and SPSS software (v25) were utilized for analyzing and interpreting data. Cox regression and Kaplan–Meier curve analyses were performed using the survival package, while the nomogram was generated utilizing the “rms” package. The ggplot2 package was utilized for all visualizations. The categorical data were subjected to a chi‐squared test, while mean ± standard deviation, percentage, or frequency were utilized to express continuous variables. The *t*‐test was employed to analyze normally distributed variables, whereas, the variables following a non‐normal distribution were analyzed utilizing the Mann–Whitney test. *p* ≤ 0.05 was taken as a statistically significant difference.

## 3 RESULTS

### 3.1 Differential expression and prognostic significance of CNIH4

**FIGURE 1:**
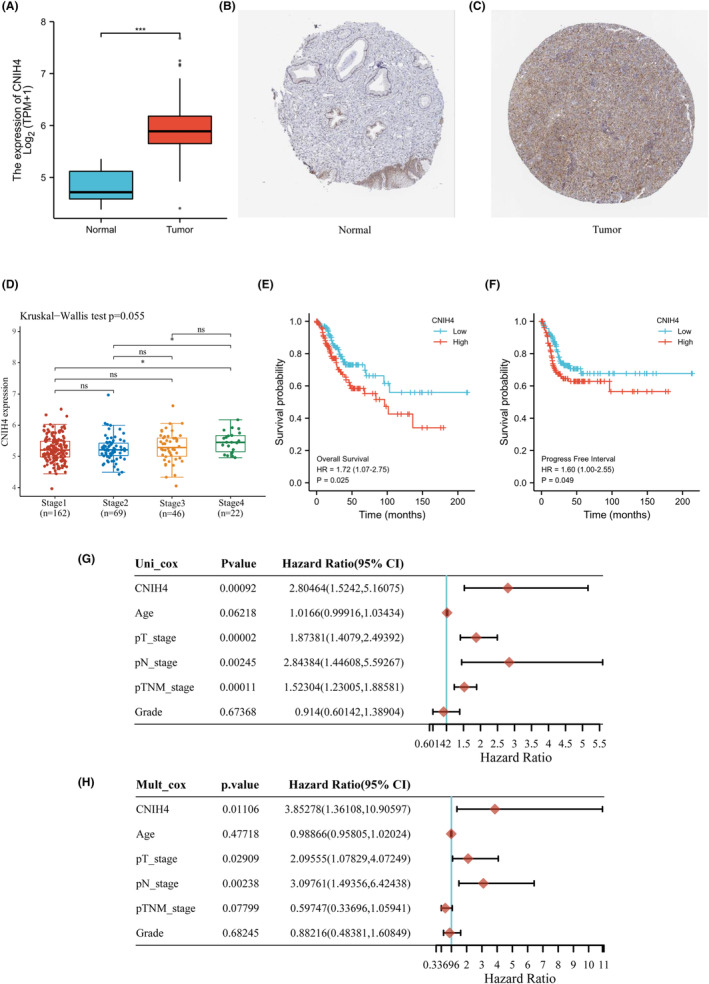
CNIH4 expression and prognosis analysis in CESC. (A) CNIH4 expression comparison in adjacent and CESC tissues. (B) CNIH4 expression in normal tissues from HPA database. (C) CNIH4 expression in CESC tissues from HPA database. (D) CNIH4 gene expression in different stage of TCGA cohort. (E) Overall Survival difference between high expression and low expression of CNIH4. (F) Progress‐free survival difference between high expression and low expression. (G) Univariate Cox regression analysis of CNIH4 in TCGA‐CESC patients. (H) Multivariate Cox regression analysis of CNIH4 in TCGA‐CESC patients. *p < 0.05, **p < 0.01, ***p < 0.001. CNIH4, cornichon homolog 4; HPA, Human Protein Atlas; TCGA, The Cancer Genome Atlas.

The expression of the CNIH4 gene in CESC tissues was considerably elevated compared to adjacent non‐tumor tissues in the TCGA‐CESC cohort (Figure 1A). CNIH4 protein expression was also elevated in CESC tissues according to the Human Protein Atlas data, which was validated by IHC staining (Figure 1B, C). Elevated gene expression of CNIH4 was linked to the advanced TNM stage (Figure 1D) and poor survival (Figure 1E,F) in the TCGA‐CESC cohort. Additionally, high expression of CNIH4 was determined as a detrimental independent prognosis factor for OS in patients with CESC, as evidenced by univariate (Figure 1G) and multivariate analyses (Figure 1H).

### 3.2 Establishing a xenograft tumor model in nude mice

**FIGURE 2:**
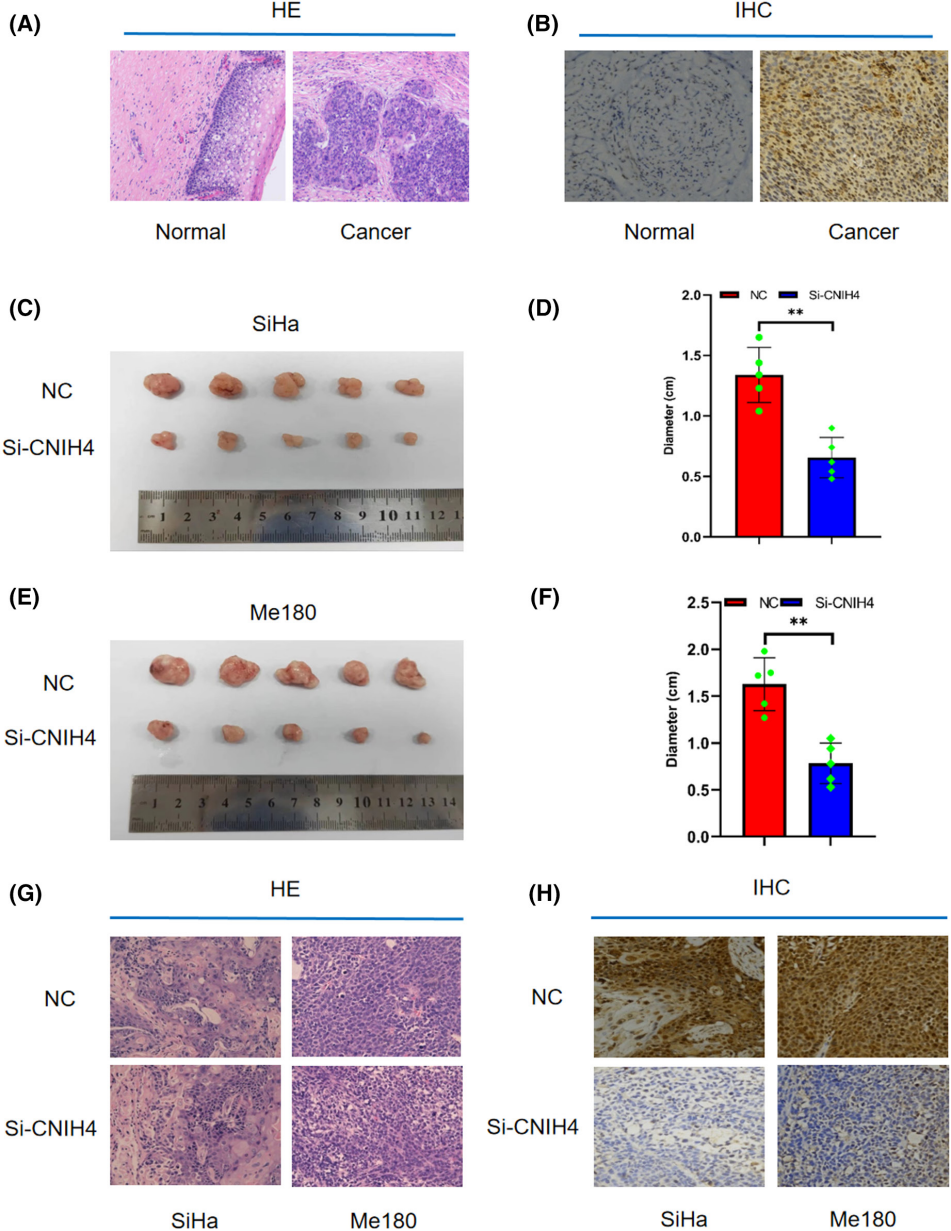
The expression of CNIH4 in CESC tissues and in SiHa and Me180 cell tumor tissues after CNIH4 knockdown. (A, B) The HE (A) and immunohistochemistry staining (B) of normal and tumor tissues (100×). (C–F) Effect of CNIH4 on tumor weight of subcutaneous transplanted tumors in SiHa (C, D) and Me180 cells (E, F) in nude mice. (G, H) The HE (G) and immunohistochemistry staining (H) of SiHa and Me180 cell tumor tissues after CNIH4 knockdown (100×). *p < 0.05, **p < 0.01, ***p < 0.001. CNIH4, cornichon homolog 4.

The expression of CNIH4 in CESC tissues was considerably elevated compared to paracancerous cervical tissues (Figure 2A,B). Tumor weights of SiHa and Me180 cell xenografted in nude mice based on CNIH4 gene knockdown were lower than those of the wild‐type nude mice (Figure 2C–F). The results of HE staining confirmed that the two established tumors were all CESCs. Compared with the wild‐type nude mice, CNIH4 expression in the knockdown group decreased significantly (Figure 2G,H), indicating that knocking down CNIH4 significantly inhibits CESC.

### 3.3 Assessing oncogenic effects of CNIH4 in CESC cells

**FIGURE 3:**
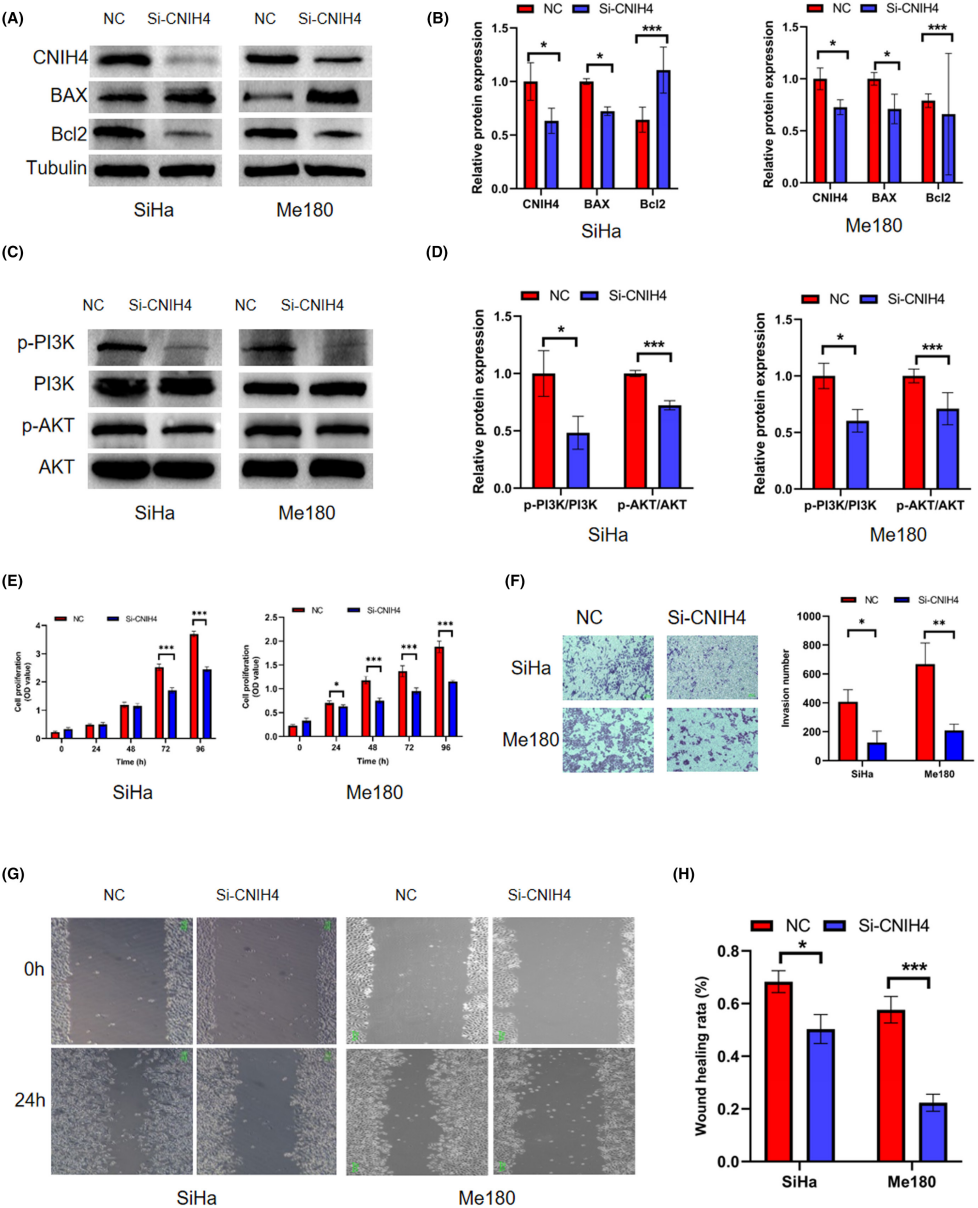
The impact of CNIH4 knockdown in CESC cell lines. (A, B) Western blot analysis of the CNIH4, BAX, and Bcl2 expression levels in SiHa and Me180 cells after CNIH4 knockdown. (C, D) WB analysis of PI3K/AKT pathway expression levels in SiHa and Me180 cells after CNIH4 knockdown. (E) The proliferation ratio of SiHa and Me180 cells from CCK‐8 assays. (F) Invasion experiments of SiHa and Me180 cells. (G, H) Migration assays of SiHa and Me180 cells. Scar bar: 100um. *p < 0.05, **p < 0.01, ***p < 0.001. CNIH4, cornichon homolog 4.

The protein expression of CNIH4 was knocked down in SiHa cells and Me180 cells using siRNA construction technology to investigate the biological significance of CNIH4 in CESC cell lines. Expressions of CNIH4, Bcl2, p‐PI3K/PI3K, and p‐AKT/AKT in the si‐CNIH4 group were significantly lower than those in the NC group, while the protein levels of the proapoptotic protein, BAX, was significantly higher compared to the NC group (Figure 3A–D). CNIH4 indeed is associated with the PI3K/AKT signaling pathway in CESC.

The results of CCK‐8 assay indicated that CNIH4 knockdown suppressed the proliferation rate of SiHa cells and Me180 cells in comparison to their respective controls (Figure 3E). In addition, siRNA‐mediated CNIH4 gene knockdown significantly inhibited cellular component (CC) cell invasion (Figure 3F) and migration (Figure 3G,H). These results indicated that CNIH4 enhances the proliferation and migration of CC cells. The data imply that it may hold promise as a prospective target for therapy in CC.

### 3.4 Functional enrichment based on RNA‐Seq sata from CESC cell lines

**FIGURE 4:**
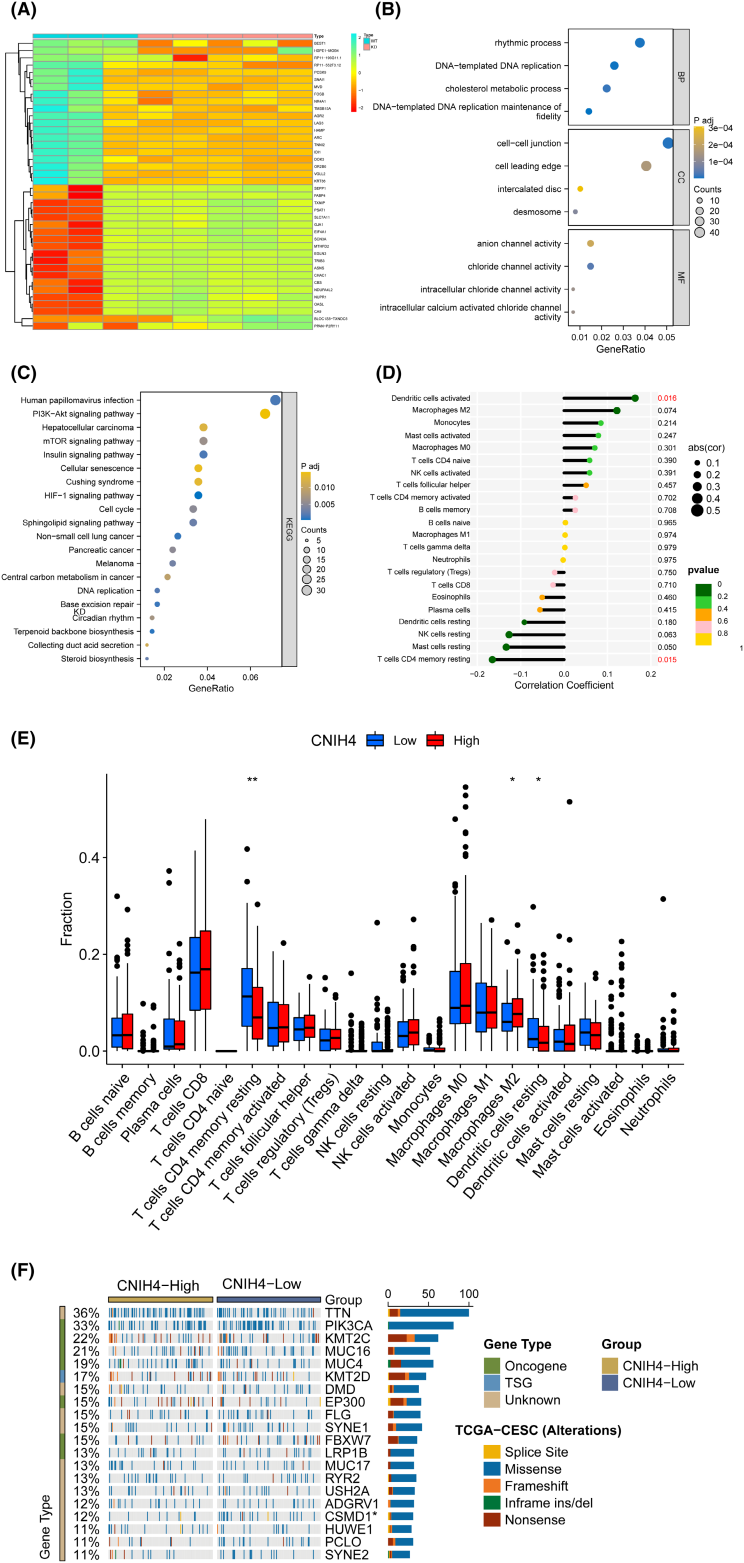
The functional enrichment analysis of RNA‐sequencing data and immune infiltration analysis. (A) The top 20 differentially expressed genes visualized by heatmap between RNA‐sequencing of knockdown and wild type of SiHa cell line. (B) GO enrichment analysis of differentially expressed genes. (C) KEGG enrichment analysis of differentially expressed genes. (D) The correlation between CNIH4 gene expression and immune cells proportions in CESC. (E) The difference of immune cells proportions between CNIH4 high‐ and low‐expression groups. (F) The difference of mutation landscape between CNIH4 low‐ and high‐ expression groups. *p < 0.05, **p < 0.01, ***p < 0.001. CNIH4, cornichon homolog 4.

To investigate the biological significance of CNIH4 in CESC, enrichment analysis was performed for DEGs associated with CNIH4, which were identified based on RNA‐Seq data obtained from the CNIH4‐knocked down SiHa cell line (Figure 4A). GO analysis demonstrated that these genes primarily participated in the in the “DNA‐templated DNA replication,” and “DNA‐templated DNA replication maintenance of fidelity” in biological process (BP); “cell–cell junction” in CC; “anion channel activity,” “chloride channel activity,” and “intracellular chloride channel activity” in molecular function (Figure 4B). KEGG analysis found that the “PI3K‐Akt signaling pathway,” “mTOR signaling pathway,” “HIF‐1 signaling pathway,” “DNA replication,” “Base excision repair,” and “Cell cycle” was substantially associated with CNIH4 in CESC (Figure 4C). These findings indicated that CNIH4 was involved in CESC tumorigenesis.

### 3.5 CNIH4 is correlated with various TIICs in CESC

To assess the influence of CNIH4 expression on the TIIC landscape in CESC tissues, a CIBERSORT deconvolution analysis was conducted. Activated dendritic cells (*r* = 0.16, *p* = 0.016) and resting memory CD4^+^ T cells (*r* = −0.17, *p* = 0.015) (Figure 4D) were significantly associated with CNIH4 in CESC samples. Comparative infiltration level of M2 macrophages was significantly elevated in the group with high CNIH4 expression, while that of and resting memory CD4^+^ T cells and resting dendritic cells were considerably decreased in the high CNIH4 expression group (Figure 4E). CNIH4 could perturb M2 macrophages, CD4^+^ T cells, and dendritic cells in CESC. The frequency of mutations in the CUB and Sushi multiple domains 1 (CSMD1) genes in the high‐expression group increased significantly compared with the low‐expression group (Figure 4F). In a previous study, CSMD1‐mut in cancer was found to be associated with increased TMB and favorable survival. This result may partly explain the relatively better prognosis of patients in the CNIH4 low‐expression group.

### 3.6 CNIH4 shows negative correlations with immune checkpoints and scores of immune pathways

**FIGURE 5:**
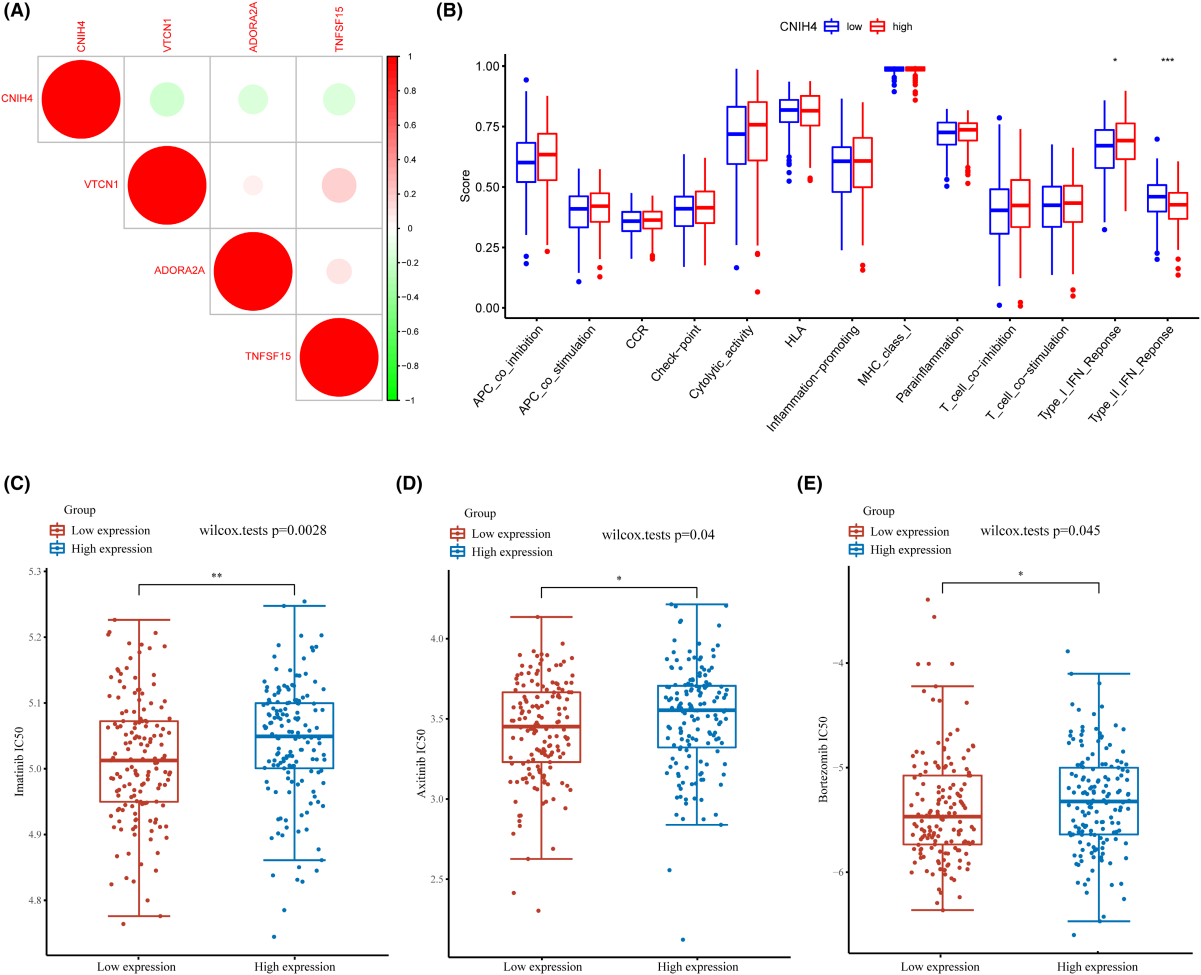
Analyses of immune checkpoints and drug therapy for CNIH4. (A) The correlation between CNIH4 and immune checkpoints in CESC. (B) Single‐sample gene set enrichment analysis. (C–E) Sensitivity of CNIH4 high‐ and low‐expression groups of TCGA‐CESC cohort to drug therapy. *p < 0.05, **p < 0.01, ***p < 0.001. CNIH4, cornichon homolog 4; TCGA, The Cancer Genome Atlas.

The gene expression of CNIH4 depicted a negative correlation with the immune checkpoints, including VTCN1, ADORA2A, and TNFSF15 (Figure 5A). Lower scores for Type‐1 and Type‐2 IFN responses were observed in the group with low CNIH4 expression in contrast to the group with high expression (Figure 5B). CNIH4 was strongly linked to the intratumoral immune landscape in CESC.

### 3.7 Evaluation of correlation of CNIH4 expression with the drug responses

GDSC was employed to explore the correlation between CNIH4 and drug therapy sensitivity and evaluate the ability of differential CNIH4 expression in discriminating drug therapeutic responses in patients with CESC. According to these findings, better therapy outcomes were observed in individuals with low CNIH4 expression than in those with high CNIH4 expression (Figure 5C–E).

### 3.8 Prognosis model based on four CNIH4‐related immunomodulators

**FIGURE 6:**
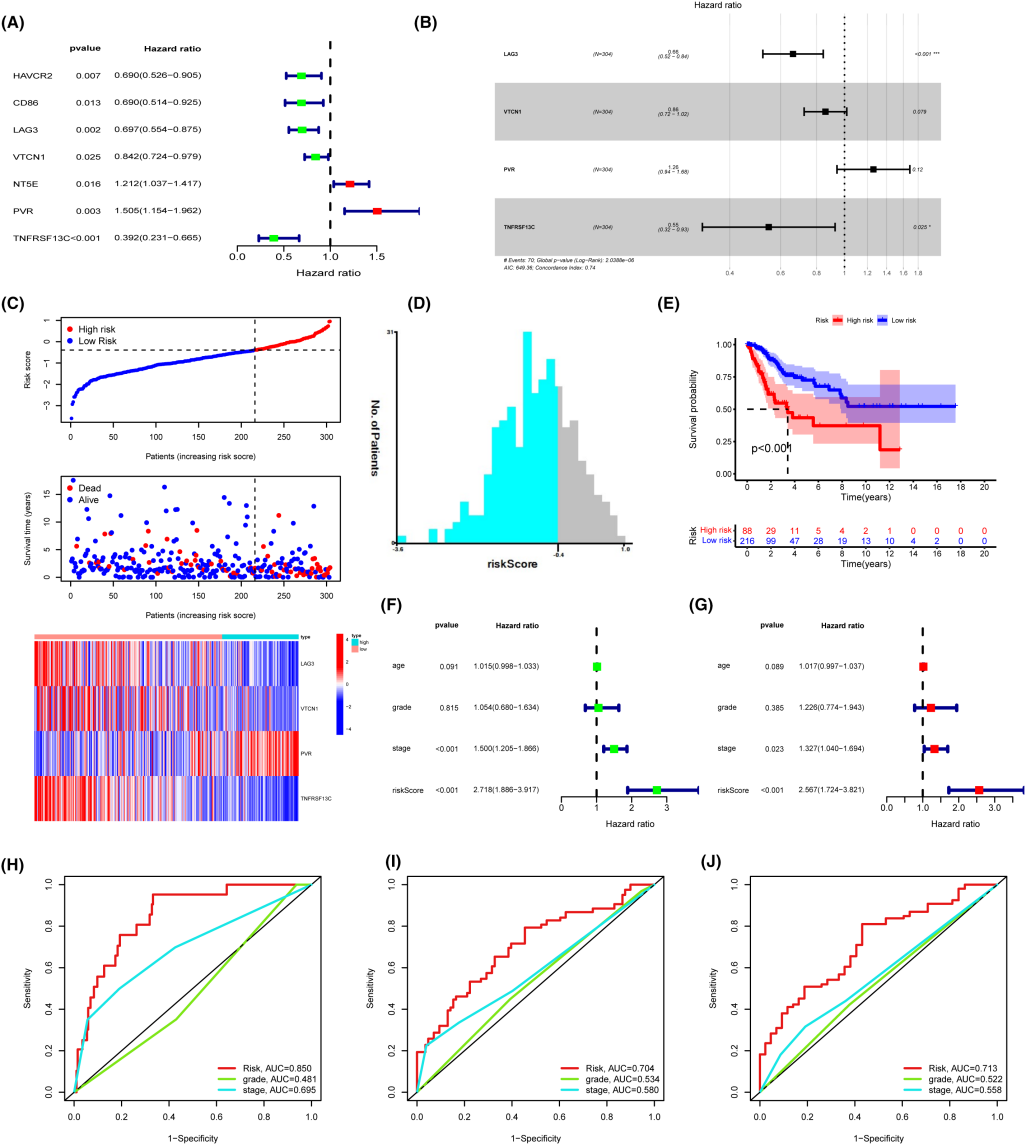
Prognostic values of the CNIH4‐revelant predictive model in TCGA‐CESC cohort. (A) Univariate Cox regression analysis of CNIH4‐related immunomodulators. (B) Forest plot exhibiting the multivariate Cox regression results. (C) Risk score, survival status and expression of CNIH4‐related immunomodulators in high‐risk and low‐risk groups of TCGA‐CESC cohort. (D) The optimal cutoff of predictive mode calculated by X‐tile software. (E) Overall survival (OS) analysis in high‐risk and low‐risk groups of TCGA‐CESC cohort. (F) Univariate Cox regression analysis of riskscore in TCGA‐CESC patients. (G) Multivariate Cox regression analysis of riskscore in TCGA‐CESC patients. (H–J) ROC analyses of 1 (H), 3 (I) and 5 years (J) OS in TCGA‐LIHC cohort. *p < 0.05, **p < 0.01, ***p < 0.001. CNIH4, cornichon homolog 4; TCGA, The Cancer Genome Atlas.

Consequently, the sum of 22 immunomodulators presented a significant association with CNIH4 in TCGA‐CESC (Spearman correlation test, *p* < 0.05, Figure S1). Seven immunomodulators were identified as substantial prognosis variables for OS in patients with CESC (Figure 6A). The identified prognosis variables were subjected to a stepwise multivariate Cox regression analysis, leading to the development of a model based on four CNIH4‐related immunomodulators (Figure 6B). The riskscore of immune genes related to CNIH4 was calculated utilizing the following equation: Riskscore = −0.4113 × LAG3 expression—0.1524 × VTCN1 expression + 0.2306 × PVR expression—0.6053 × TNFRSF13C expression (Figure 6C). The optimal cutoff was set to −0.39 (Figure 6D). Kaplan–Meier curves demonstrated that the prognosis of individuals at high risk was poor (Figure 6E). The risk score was unveiled as an adversely independent prognosis factor for patients with CESC (Figure 6F,G). AUC was utilized to assess the efficiency of risk scores for predicting the 1‐ and 3‐year OS rates of TCGA‐CESC patients. The respective values for the 1‐, 3‐, and 5‐year AUC were 0.850, 0.704, and 0.713, which showed superior accuracy in prediction efficacy compared with TNM stage and histopathological grade classifier (Figure 6H–J).

## 4 DISCUSSION

Globally, CESC ranks fourth in incidence among women after breast, colon, and lung cancers.^11^ Additionally, it is the primary cause of cancer‐related fatalities among women. Since the origin and progression of CESC is a complex biological process involving multiple molecular regulators, the detection of molecular‐based biomarkers is expected to benefit the development of individualized therapy for patients with CESC.

Our results demonstrated that CNIH4 was overexpressed in CESC tissues at both gene and protein levels. Elevated CNIH4 gene expression could independently predict poor OS in patients with CESC. siRNA‐mediated CNIH4 knockdown contributed to decreased cell proliferation, invasion, and migration in SiHa and Me180 cell lines. Knocking down CNIH4 significantly suppresses tumor growth as demonstrated in a nude mouse xenograft tumor model, providing evidence for the involvement of upregulated CNIH4 expression in the development of CESC.

GO and KEGG enrichment revealed that CNIH4 was involved in extracellular matrix‐related cell–cell junction, mTOR signaling, PI3K‐AKT signaling, and TGF‐β signaling pathways in CESC. These pathways are closely related to malignancy in CNIH4. For instance, PI3K‐AKT and mTOR signaling pathways are critically involved in tumor invasion and metastasis.^12^, ^13^, ^14^, ^15^ The PI3K‐AKT pathway mediates the development and differentiation of both T and B cell populations.^16^, ^17^ Inhibition of the PI3K pathway could elevate the infiltration of CD8^+^ T cells and regulatory T cells into tumor tissues, regulate immunosuppressive cytokine release, and promote memory T‐cell formation.^18^, ^19^ The interaction between these biological processes and CNIH4 may be important in the malignant progression of CESC.

Regarding the impact of CNIH4 expression on the intratumoral immune landscape, the study found that CNIH4 was linked to dendritic cells, CD4^+^ T cells, and M2 macrophages. CD4^+^ memory T cells are essential for effectively controlling immune function, including tumor immunosurveillance, and positively impacting the prognosis of patients with CESC.^20^ The tumor‐associated macrophage phenotype, M2, can contribute to tumor angiogenesis by regulating angiogenesis factors, including VEGF and IL‐8, and is linked to poor prognosis.^21^ High CNIH4 expression may elevate M2 phenotype infiltration and reduce the proportion of memory CD4^+^ T cells, leading to a poor prognosis in CESC. However, such precise regulatory mechanisms of CNIH4 expression in tumor immune microenvironment need to be elucidated in further studies. Furthermore, patients in the CNIH4 high‐expression group exhibited low‐immunity activity, including Type‐I IFN and Type‐II IFN responses. Inhibition of Type‐I IFN signaling in tumors could mediate resistance to ICB treatment.^22^ Tumor‐infiltrating regulatory T cells (Tregs) permit tumor growth and neuropilin‐1 is needed to maintain the stability and function of the intratumoral Tregs. Type‐II IFN could promote tumor responses to immune checkpoint blockade immunotherapy by inducing the generation of intratumoral neuropilin‐1‐deficient Tregs.^23^ ssGSEA results revealed that patients with low CNIH4 expression could benefit more from immune therapy compared to the patients with high CNIH4 expression.

Chemotherapy and targeted therapy are common treatment options in clinical settings. Therefore, the sensitivity of patients with CESC to commonly prescribed drugs for chemotherapy and targeted therapy was compared between different groups based on the expression of CNIH4. The findings demonstrated that the group with reduced expression showed a higher sensitivity to chemotherapy and targeted drugs. The abovementioned results of functional enrichment analysis showed that the extracellular matrix interaction was linked to CNIH4 in CESC. Alteration in extracellular matrix stiffness, a barrier, could hinder the uptake and spread of small molecule drugs in the local tumor microenvironment of cancer cells and subsequently affect drug sensitivity in tumors. Biological processes through which CNIH4 is involved in CESC may result in differential drug sensitivity between the groups.

Given that several gene signatures have been previously identified as predictors of outcomes in many cancers, and the observed correlation between CNIH4 and the intratumoral immune landscape, this study aimed to evaluate whether intratumoral immune markers related to CNIH4 could be used to construct a prognostic model for patients with CESC. Because immunomodulators can influence tumor escape and immune destruction across cancer disease, CNIH4‐related immunomodulators were utilized to establish a prediction model. The present study constructed a prediction model utilizing a panel of four immune genes, including LAG3, VTCN1, PVR, and TNFRSF13C. LAG3, VTCN1, and TNFRSF13C were protective factors for OS in patients with CESC, whereas PVR adversely affected survival.

Despite the comprehensive clarification of the tumorigenic effect of CNIH4 in CESC, which has not been previously reported, this study has certain limitations that warrant consideration. As it relied mainly on online databases, further experimental validation in an independent patient cohort is necessary. Furthermore, additional scientific study is needed to reveal how CESC cells recruit in the case of dysregulation of CNIH4 expression.

The developed model demonstrated a strong ability to predict prognosis, with superior performance compared to the clinical TNM characteristics. As a four‐marker signature was used to generate this model, it was more inconvenient for clinical application than the published immune‐related molecule models that were constructed utilizing at least 10 variables.^24^, ^25^ Overall, this predictive model associated with the molecular landscape of CESC could supplement the TNM staging system in predicting the prognosis of patients with CESC. Additionally, it may further enhance the prognostic value of CNIH4 expression in CESC.

## 5 CONCLUSION

In summary, CNIH4 is markedly upregulated in cervical cancer and its high expression correlates with a poor prognosis. In vitro and vivo assays demonstrated that CNIH4 accelerates the proliferation and migration of cervical cancer cells by activating the PI3K‐Akt pathway.

## AUTHOR CONTRIBUTIONS

**Jiajia Wang:** Data curation (lead); investigation (equal); methodology (equal); validation (lead); visualization (lead); writing – original draft (lead); writing – review and editing (lead). **Shudan Wang:** Data curation (equal); validation (equal). **Junli Wang:** Methodology (equal). **Jingjing Huang:** Data curation (equal); writing – original draft (equal). **Haishan Lu:** Data curation (equal); visualization (equal). **Bin Pan:** Data curation (equal); methodology (equal); visualization (equal). **Hanyi Pan:** Data curation (equal); investigation (equal). **Yanlun Song:** Software (equal); visualization (equal). **Qianqian Deng:** Data curation (equal); visualization (equal). **Xiaojun Jin:** Conceptualization (lead); funding acquisition (lead); methodology (lead); resources (lead); supervision (lead). **Guiling Shi:** Conceptualization (lead); methodology (lead); resources (lead); supervision (lead).

## FUNDING INFORMATION

This work was supported by 2023 Innovation Project of Youjiang Medical University for Nationalities Graduate Education (YZCXJH2023006).

## CONFLICT OF INTEREST STATEMENT

The authors declare no conflict of interest.

## ETHICS STATEMENT

In review Human cervical cancer tissue and non‐tumor cervical tissue were obtained from patients who were admitted to Department of Obstetrics and Gynecology, The Affiliated Hospital of Youjiang Medical University for Nationalities, China in 2022. The project was approved by the Ethics Committee of the Affiliated Hospital of Youjiang Medical University for Nationalities and written informed consent was obtained from each patient that was enrolled in the study. All patients consent to publication.

## Supporting information


Figure S1.

## Data Availability

The data were downloaded from the TCGA database (https://www.cancer.gov/CESCg/research/genome‐sequencing/tcga) and CAMOIP online website (https://www.camoip.net). The Genomics of Drug Sensitivity in Cancer database (https://www.cancerrxgene.org/).
