# Correction to “Comprehensive analysis of clinical prognosis and biological significance of CNIH4 in cervical cancer”Wang J, Wang S, Wang J, Huang J, Lu H, Pan B, Pan H, Song Y, Deng Q, Jin X, Shi G. Comprehensive analysis of clinical prognosis and biological significance of CNIH4 in cervical cancer. Cancer Med. 2023;12(24):22381–22394. doi: 10.1002/cam4.6734

**DOI:** 10.1002/cam4.70214

**Published:** 2024-09-08

In section 2.7 on page 22383, “Tubulin (1:20000, Proteintech)” should have read “Actin (1:80000, ABclonal).” In Figure 3, Part A, on page 22388, “Tubulin” was incorrect and should have read “Actin.” Please see the corrected figure below. Although we used both Actin and Tubulin as the internal reference genes in the pre‐experiment, the statistics of the data show Actin instead of Tubulin.

**Figure 3:**
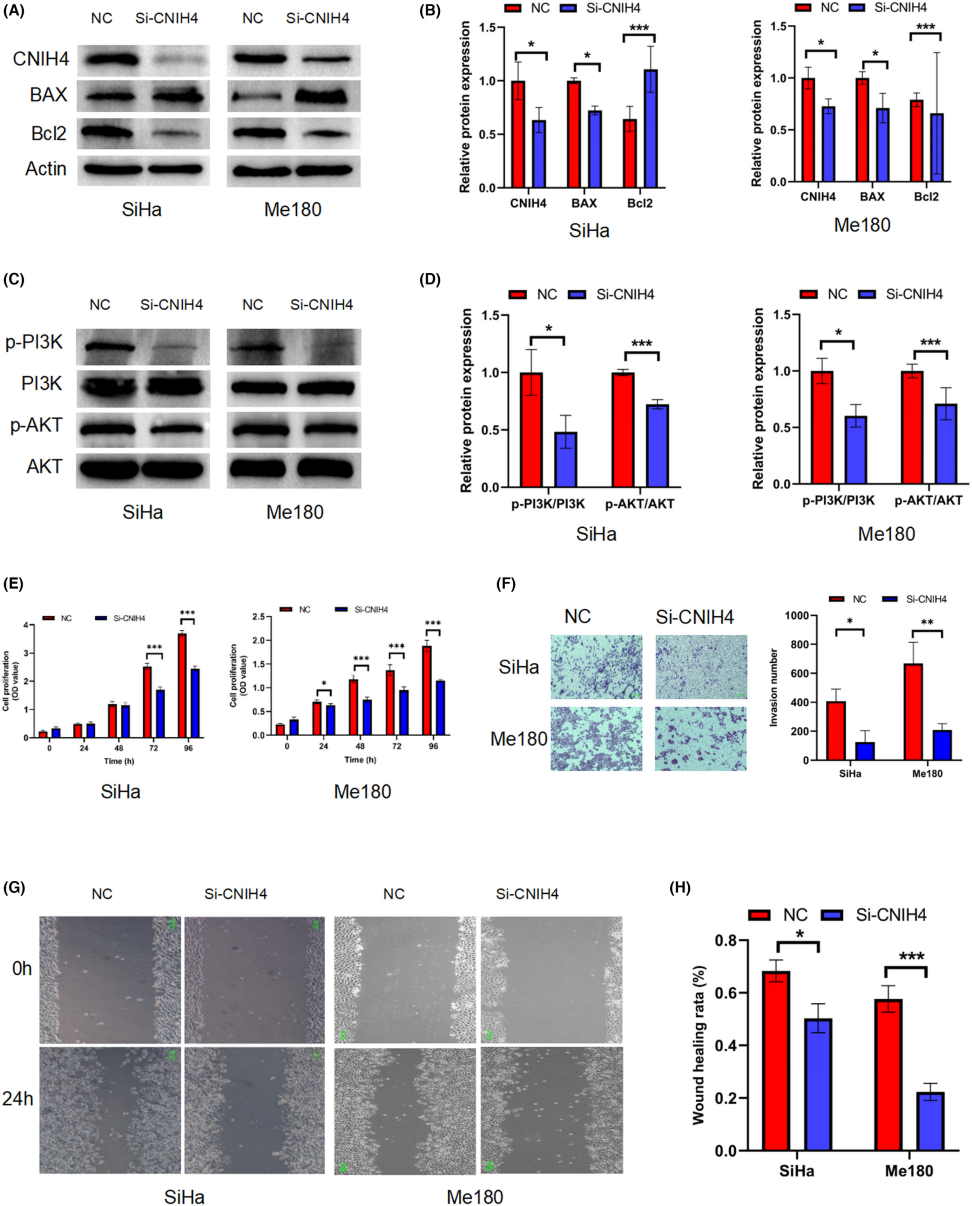


We apologize for these errors.

