# Supplementary figures and images for: Comprehensive analysis of clinical prognosis and biological significance of CNIH4 in cervical cancer
Source: Cancer Med. 2023 Dec 12;12(24):22381–94. doi: 10.1002/cam4.6734 (PMC10757085; doi:10.1002/cam4.6734)

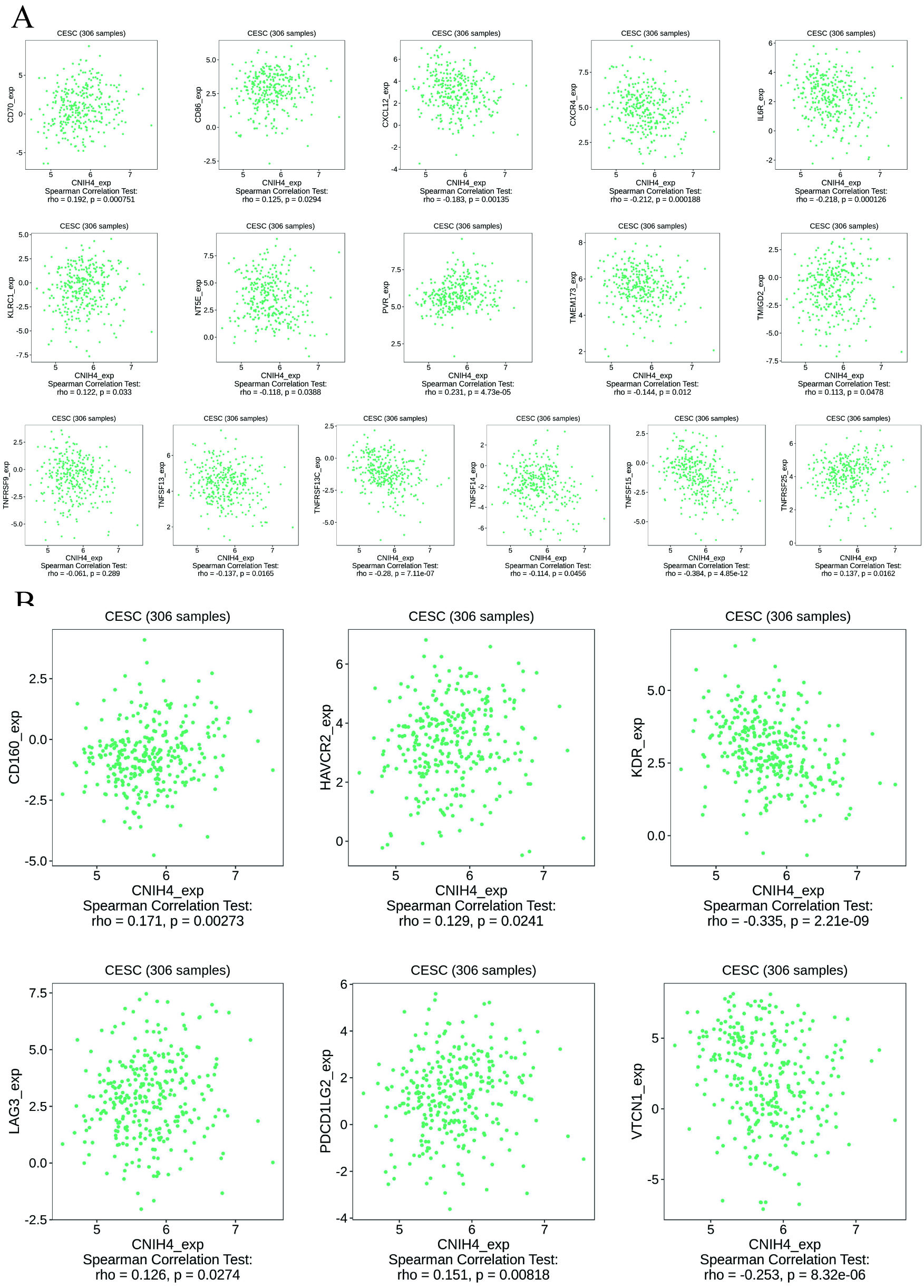

Supplement: Supplementary file 1 — Figure S1. [file CAM4-12-22381-s001.tif]
